# Socioeconomic inequalities in 29 childhood diseases: evidence from a 1,500,000 children population retrospective study

**DOI:** 10.1186/s12889-021-11230-9

**Published:** 2021-06-16

**Authors:** Neus Carrilero, Albert Dalmau-Bueno, Anna García-Altés

**Affiliations:** 1 Agència de Qualitat i Avaluació Sanitàries de Catalunya (AQuAS), Carrer de Roc Boronat, 81-95, 08005 Barcelona, Spain; 2 Universitat Pompeu Fabra. Department of Experimental and Health Sciences (DCEXS), Barcelona, Spain; 3 Institut de Recerca de l’Hospital de la Santa Creu i Sant Pau, Barcelona, Spain; 4 CIBER de Epidemiología y Salud Pública (CIBERESP), Barcelona, Spain; 5 Institut d’Investigació Biomèdica (IIB Sant Pau), Barcelona, Spain

**Keywords:** Health inequalities, Socioeconomic position, Gender, Childhood diseases, Health status disparaties, Child

## Abstract

**Background:**

Socioeconomic position (SEP) powerfully affects health status in the childhood population. However, the knowledge of which diseases are more affected by SEP and whose outcomes could be improved by having a more equitable society remains uncertain on a population basis.

**Methods:**

We measured socioeconomic and gender inequalities in the pre-COVID-19 era for 29 diseases in the entire childhood population in Catalonia to identify which diseases are most impacted by inequalities. This population-based study included 1,449,816 children under 15 years old from 2014 to 2017 (48.52% girls) and each of their registered diagnoses within the Catalonia National Health System. We calculated frequency measures by SEP and their sex ratios for each disease. We estimated four regression-based inequality measures: slope index of inequality, relative index of inequality (RII), absolute population-attributable fraction, and population-attributable fraction.

**Results:**

Twenty-five of the 29 diseases examined showed SEP inequalities. The diseases with the greatest inequalities in both sexes were tuberculosis, obesity, adjustment and anxiety disorders, essential hypertension, poisoning, short gestation, low birth weight, foetal growth retardation and intrauterine hypoxia and birth asphyxia and trauma (RII ≥ 2.0); only food allergy showed the opposite pattern (RII < 1.0). Overall, 80,188 (7.80%) of the disease events in boys and 74,921 (8.88%) in girls would be avoided if all children had the same disease rate as those in the medium-high SEP group, with tuberculosis, intrauterine hypoxia and birth asphyxia and trauma, obesity, and short gestation, low birth weight, foetal growth retardation being those that could be reduced the most in relative terms, and dermatitis, injuries, acute bronquitis, and being overweight those that could be reduced the most in absolute terms. Girls present higher RII than boys for respiratory allergy, asthma, dermatitis, being overweight, and obesity (*p* < 0.05). In contrast, boys showed higher RII compared to girls only in congenital anomalies (*p* < 0.05).

**Conclusions:**

Socioeconomic and gender inequalities are widely present in childhood health. This indicates that SEP plays a common role in their development although it varies in magnitude according to each disease. It is also a phenomenon that comprises all SEP groups in society. Action needs to be taken to ensure a fairer start in life in terms of health.

**Supplementary Information:**

The online version contains supplementary material available at 10.1186/s12889-021-11230-9.

## Background

Children are one of the most vulnerable population groups, since they are entirely dependent on their environment and have few mechanisms to face adversity. Evidence suggests that beyond biological factors, social determinants (i.e household resources, parents’ behaviours, neighbourhood deprivation, among others) [1] play an important role in children’s health status since social determinants have been systematically associated with a wide variety of pathologies of different etiologies and also in diverse societies [2].

The Early Child Development Framework (proposed by the WHO (TEAM-ECD)) highlights two essential inputs to be taken into account in regard to the production of inequalities in children’s health: (1) the stage of development in the early years of childhood, and (2) the socioeconomic position (SEP) of the child’s family [3, 4]. It is the family context that provides the most influence on the child and which also controls the inputs received from the distal environment [1]. Previous studies have established a “gradient effect” according to the family’s SEP across a wide range of outcomes, both in childhood and adulthood, in health, educative attaintment, and in future career opportunities [5–9]. Therefore, tackling inequalities at the beginning of life and focusing on improving family support is a powerful approach to establishing the roots of good health in childhood development and in the future to come [2–4, 9].

Catalonia’s situation, as in other European countries, is far from achieving the Sustainable Development Goal (SDG) 10 of “Reduced inequality” [10]. In Catalonia, poverty and social exclusion rates in childhood have been increasing since 2008 reaching an at-risk-of-poverty or social exclusion rate of 33.0% in 2019, far above the European average of 22.5%. Besides, children in Catalonia suffer the most from poverty, more than other age groups [11, 12]. In spite of this high poverty rate, macro health indicators such as life expectancy at birth and infant mortality have improved since 1994, maintaining a similar downward trend as is seen in other European countries [13, 14]. By contrast, poor health outcomes related to social determinants and lifestyle are indicators that have worsened in Catalonia and other similar contexts; these indicators include low birth weight rate, being overweight, higher consumption of sugary beverages, and physical activity engagement, among others [14–18].

Socioeconomic inequalities in these outcomes have also widened [19], as well as indicators of healthcare use among the youngest population [20].

Despite the growing interest in health inequalities, thus far, childhood diseases have mainly been analysed individually with different socioeconomic proxies, populations, contexts, and inequality measures [6]. These different approaches do not contribute to an accurate comparison between inequality measures or between health outcomes. A parallell but equally important issue is that gender differences in health inequalities have been scarcely analyzed [21]. It is increasingly recognized that different axes of social power relations such as gender and SEP, are interrelated impacting on health in an intersecting process [21–24]. It is strongly recomended that SEP should not be analysed by itself because apparent inequalities can be misinterpreted without gender analysis. It could open a wider area of research in inequalities and gender since the genderization of health determinants already starts in childhood [22, 23].

Therefore, the aim of this study is threefold: 1) to calculate socioeconomic inequality for a wide range of diseases in an entire childhood population, 2) to determine gender differences in inequalities in each disease, and 3) to estimate the burden of disease attributable to SEP using public health impact measures. To accomplish these aims, we analysed 29 diseases by sex using summary inequality and impact measures that are widely used in public health epidemiology based on regression models that quantify inequality and population impact including all population SEP levels, rather than the two extreme groups [25, 26].

## Methods

Healthcare in Catalonia is organised as a National Health System, funded by taxes. All residents (7,348,275 as of 2017) are granted universal public healthcare coverage by law. The use of publicly funded healthcare services is free; the sole exception is drug prescription, which is based on a co-payment system. Each resident is assigned a unique personal healthcare ID which can be used to trace their use of healthcare services.

### Study design and population

We carried out a population-based study on a retrospective review of all children under 15 resident in Catalonia during the period 2014–2017 (1,449,816 children, 48.52% girls).

### Data collection

Two different databases were used:

The Central Registry of Insured Persons (RCA, Catalan acronym), to obtain the reference population for the 2014–2017 period. This registry also collects and updates data on the annual income level, employment status, and Social Security benefits of each individual, which are routinely used to calculate citizens’ pharmaceutical copayment levels.

The Registry of the Minimum Basic Dataset (CMBD, Catalan acronym) is an administrative register containing detailed information on sociodemographic characteristics and medical diagnoses (coded using the International Classification of Diseases, 9th Edition). The CMBD encodes all contacts with the public healthcare system at an individual level: primary care, hospital care, emergency, mental health, and long-term care services. All the diagnostic codes registered in the CMBD during the period 2014–2017 were gathered to create the main dataset which was then linked to the RCA through a common personal healthcare ID.

Both registries are considered of high quality as 1) they include the entire population of Catalonia since all citizens are granted universal healthcare coverage (99.6% of all residents are registered); 2) they have a validation system; and 3) they contain historical diagnosis data. We excluded 307 children (0.02% of the entire child population) due to a misclassification of the SEP variable.

### Variables

The dependent variables were 29 diseases or risk factors representative of the health status of the child population of Catalonia. This wide selection includes both diseases that are strongly influenced by social determinants and others whose influence is not clearly established [6, 27]. This selection also represents diseases of diverse etiology, affecting different organic systems, and of differing severity. Each disease variable was constructed selecting diagnostic codes based on clinical criteria (see Additional file 1). The inclusion criteria at an individual level was having at least one medical diagnosis, being registered on the CMBD, and included in at least one of the defined diseases. Duplicate diagnoses were not taken into account so that each of the disease variables was treated as a dichotomous variable.

The independent variables were SEP, age, and sex. SEP and age were defined at the beginning of the study period. SEP was proxied by the pharmaceutical copayment level of the child’s parents or guardians, obtained from the RCA registry. Pharmaceutical copayment uses income level and employment status information (specially to detect individuals without any income from work) to define four SEP categories as follows: “Very low” (no member of the household employed or receiving welfare support), “Low” (<€18,000/year), “Medium” (€18,000–€100,000/year), and “High” (>€100,000/year). This classification has been used in previous studies [20, 28]. Age was categorised into five groups (0–2 years, 3–5 years, 6–8 years, 9–11 years, and 12–14 years) and sex (boys, girls).

### Statistical analysis

Age and SEP distribution were calculated first with absolute (N) and relative frequency (%). Following the profile of each disease (acute or chronic), we calculated for each SEP category age-adjusted incidence proportion or period prevalence. Incidence proportion was calculated only for acute bronchitis, influenza, injuries, meningitis, poisoning, and tuberculosis (TB); accounting for the new cases diagnosed in the period; period prevalence was calculated for the rest of the diseases, accounting for new and pre-existing cases. The prevalence ratios (PR) or incidence proportion ratios (IR) for boys and girls were calculated through a generalised linear model (log-binomial regression) with a logarithmic link function. Both PR and IR were estimated with their respective confidence intervals (95% CI) and associated *p*-values.

To quantify SEP inequalities in each disease, we calculated two different summary measures: the relative index of inequality (RII) and slope index of inequality (SII). These indices are regression based and accounted for the sample size population and relative weight for every stratum of SEP, rather than comparing only the two most extreme SEP levels. For both indices, the punctual estimate is accompanied by its 95% CI and the associated *p*-value.

The measure of RII is a relative measure of inequality that involves the following steps: 1) determination of the disease frequency of each SEP category and 2) ranking SEP levels, assigning a hierarchical value from 0 (lowest SEP) to 1 (highest SEP) according to its proportional size in the population by linear regression. The population of each SEP level is assigned a Ridit score based on the mid-point of the cumulative distribution of the population of participants in the given category, and finally 3) this weighted SEP is included as exposure and disease as a dichotomous dependent variable in a generalised linear model (log-binomial regression) with a logarithmic link function. The highest SEP category was used as a reference. The RII reflects the risk ratio of an outcome between those on the social hierarchy who are more advantaged and those who are more disadvantaged [29, 30].

The SII is an absolute measure of inequality that represents the absolute difference in estimated values of a health outcome between the most advantaged and the most disadvantaged. The calculation of SII follows the same steps as RII: 1) and 2) and then, 3) weighted SEP is also included as exposure and frequency of disease as a dependent variable in a generalised linear model adjusted by age with an identity link function. The difference in the predicted values for the extreme SEP categories generates the SII value. If there is no inequality, SII takes the value zero. Greater absolute values indicate higher levels of inequality [29, 30].

Finally, to study intersectionality between sex and SEP across diseases, sex differences were assessed by inserting a two-way interaction term between the SEP Ridit score calculated previously and gender for the RII and SII models. A positive and significant coefficient for the two-way interaction term would indicate a larger increase in RII or SII in girls compared to boys [31].

We calculated population attributable fractions in absolute terms (PAFa) and relative terms (PAF%). To achieve this, medium and high SEP categories were grouped and defined as the reference group and low and very low SEP as the exposure group; the log-binomial model mentioned previously were performed again. PAFa are the estimated cases that would be prevented if low and very low SEP had the same health status as higher SEP levels while other risk factors remain unchanged. PAF% is the associated fraction and its PAF% = pd.[(PRa-1)/PRa], where PRa is the prevalence ratio, pd. is the proportion of those with the specific disease and PRa-1 represents the risk over the reference point. For incidence proportion substitute PRa for IRa. An example of interpretation is, for instance: TB in girls has a PAF% = 51.95% of the total TB events in girls which could be potentially avoided if all the population had the same TB incidence as medium-high SEP, which is translated in PAFa = 171 TB events. The PAFa and PAF% results are presented with a 95%CI and the associated *p*-values [25, 29, 32].

All analyses were performed separately for girls and boys. All methods were carried out in accordance with relevant guidelines and regulations. We used the STATA IC/15.1 software modules RIIGEN for RII and SII and Punaf for PAF, to perform all the analyses [33, 34].

## Results

The characteristics of the study population are presented in Table 1. During the study period, the population contained 1,449,816 children under 15 years old (48.52% girls and 51.48% boys); the largest group was of those aged from newborn to 2 years representing around 30% of boys and girls. In our population, 4.21% (60,994 children) are very low SEP (4.24% in boys and 4.17% in girls), and the low SEP represents the main group (62.39% in boys and 62.30% in girls).

**Table 1:** Characteristics of the child population by SEP and age^a^ in Catalonia during the period 2014–2017

	Boys		Girls		Total	
	*N* = 746,392	%	*N* = 703,424	%	*N* = 1,449,816	%
**Age**						
0–2	224,889	30.13	211,827	30.11	436,716	30.12
3–5	140,194	18.78	131,100	18.64	271,294	18.71
6–8	135,725	18.18	127,797	18.17	263,522	18.18
9–11	127,066	17.02	120,194	17.09	247,260	17.06
12–14	118,518	15.89	112,506	15.99	231,024	15.93
**SEP**						
Very low	31,641	4.24	29,353	4.17	60,994	4.21
Low	465,703	62.39	438,256	62.30	903,959	62.35
Medium	243,055	32.57	230,005	32.70	473,060	32.63
High	5993	0.80	5810	0.83	11,803	0.81

^a^SEP and age were determined for the first year of the study period

### Prevalences, incidence proportions, PR and IR

Prevalences, incidence proportions by sex and SEP, and PR and IR between sexes for each disease are shown in Table 2. The most frequent diseases or risk factors in both sexes were injuries (34.51% in boys and 29.25% in girls), dermatitis (21.22% in boys and 23.30% in girls), and acute bronchitis (18.32% in boys and 15.23% in girls). Congenital anomalies, being overweight, influenza, chronic bronchitis, obesity, and asthma had proportions between 5 and 10% in both sexes.

**Table 2:** Disease frequency measures by SEP and sex, and Prevalence ratio (PR) or Incidence proportion ratio (IR) (boys compared to girls) of children in Catalonia

	PR/IR	Period prevalence/ Incidence proportion							
	Boys vs girls	Boys				Girls			
		Very low SEP	Low SEP	Medium SEP	High SEP	Very low SEP	Low SEP	Medium SEP	High SEP
Disease	PR/IR (95% CI) *p*-value	N (%)	N (%)	N (%)	N (%)	N (%)	N (%)	N (%)	N (%)
Infectious diseases									
Tuberculosis^a^	0.92 (0.79, 1.07)	33 (0.10)	243 (0.05)	47 (0.02)	0 (0.00)	44 (0.15)	229 (0.05)	53 (0.02)	0 (0.00)
Meningitis (bacterial or viral)^a^	1.57 (1.36, 1.80)***	33 (0.10)	330 (0.07)	142 (0.06)	1 (0.02)	12 (0.04)	196 (0.04)	97 (0.04)	1 (0.01)
Influenza^a^	1.05 (1.04, 1.06)***	3257 (10.29)	40,304 (8.65)	19,581 (8.06)	234 (3.91)	2940 (10.02)	36,172 (8.25)	17,580 (7.64)	217 (3.73)
Neoplasms									
Cancer of the brain and nervous system	1.09 (0.90, 1.32)	18 (0.06)	133 (0.03)	75 (0.03)	0 (0.00)	6 (0.02)	126 (0.03)	58 (0.03)	4 (0.06)
Leukaemia	1.26 (1.06, 1.48)**	17 (0.05)	196 (0.04)	105 (0.04)	4 (0.07)	8 (0.03)	157 (0.04)	74 (0.03)	1 (0.02)
Malignant neoplasms	1.08 (1.02, 1.15)*	129 (0.41)	1402 (0.30)	667 (0.27)	9 (0.15)	107 (0.36)	1231 (0.28)	572 (0.259	8 (0.14)
Anthropometrics									
Overweight	1.06 (1.05, 1.08)***	3809 (18.77)	44,974 (17.52)	20,602 (14.23)	267 (5.93)	3252 (17.24)	38,425 (15.80)	16,985 (12.30)	228 (5.30)
Obesity	1.33 (1.32, 1.35)***	2159 (10.64)	23,454 (9.14)	8886 (6.14)	78 (1.73)	1530 (8.11)	16,237 (6.71)	5880 (4.27)	57 (1.32)
Mental health									
Mood disorders	0.87 (0.83, 0.91)***	164 (0.52)	2144 (0.46)	780 (0.32)	10 (0.17)	200 (0.68)	2325 (0.53)	823 (0.36)	10 (0.17)
Adjustment and anxiety disorders	1.06 (1.04, 1.08)***	1788 (5.65)	21,098 (4.53)	7596 (3.13)	56 (0.94)	1638 (5.58)	18,764 (4.28)	6682 (2.91)	47 (0.82)
ADHD	2.70 (2.63, 2.76)***	1323 (4.18)	16,443 (3.53)	7790 (3.20)	139 (2.32)	437 (1.49)	5863 (1.34)	2627 (1.14)	46 (0.79)
ASD	4.66 (4.39, 4.94)***	321 (1.01)	4154 (0.89)	2006 (0.83)	21 (0.35)	68 (0.23)	844 (0.19)	401 (0.17)	2 (0.04)
Autoimmune and respiratory diseases									
Acute bronchitis^a^	1.21 (1.20, 1.21)***	7014 (22.17)	87,062 (18.60)	41,963 (17.26)	669 (11.16)	5471 (18.64)	68,208 (15.50)	32,929 (14.30)	476 (8.20)
Chronic bronchitis	1.22 (1.21, 1.23)***	3285 (10.38)	40.515 (8.70)	19,404 (7.98)	282 (4.71)	2560 (8.72)	31,430 (7.17)	14,976 (6.51)	201 (3.46)
Asthma	1.47 (1.45, 1.49)***	2460 (7.77)	32,359 (6.95)	16,639 (6.85)	232 (3.87)	1641 (5.59)	21,122 (4.82)	10,298 (4.48)	128 (2.20)
Respiratory allergy	1.13 (1.12, 1.14)***	1677 (5.30)	22,128 (4.75)	9797 (4.03)	125 (2.09)	1310 (4.46)	15,929 (3.63)	6640 (2.89)	79 (1.36)
Dermatitis	0.94 (0.93, 0.94)***	7610 (24.05)	102,065 (21.92)	47,832 (19.68)	668 (11.15)	7886 (26.87)	106,201 (24.23)	48,956 (21.28)	687 (11.82)
Food allergy	1.08 (1.06, 1.10)***	316 (1.00)	5502 (1.18)	3811 (1.57)	58 (0.96)	240 (0.82)	5073 (1.16)	3640 (1.58)	59 (1.01)
Cystic fibrosis	1.00 (0.79, 1.28)	3 (0.01)	83 (0.02)	46 (0.02)	1 (0.01)	5 (0.02)	75 (0.02)	44 (0.02)	2 (0.04)
Adverse birth outcomes									
Short gestation, low birth weight, foetal growth retardation	1.04 (1.01, 1.08)*	320 (1.01)	4795 (1.03)	1677 (0.69)	30 (0.50)	310 (1.05)	4276 (0.98)	1568 (0.68)	28 (0.49)
Intrauterine hypoxia and birth asphyxia and trauma	1.37 (1.29, 1.45)***	173 (0.55)	2102 (0.45)	746 (0.31)	8 (0.14)	85 (0.29)	1480 (0.34)	506 (0.22)	0 (0.00)
Congenital anomalies	1.18 (1.16, 1.19)***	3343 (10.57)	46,752 (10.04)	21,640 (8.90)	265 (4.42)	2398 (8.17)	36,981 (8.44)	18,183 (7.91)	232 (3.99)
Nervous system									
Paralysis	1.24 (1.14, 1.36)***	71 (0.22)	760 (0.16)	306 (0.13)	3 (0.05)	64 (0.22)	544 (0.12)	252 (0.11)	4 (0.07)
Epilepsy	1.16 (1.10, 1.21)***	217 (0.68)	2555 (0.55)	1139 (0.47)	28 (0.46)	175 (0.60)	2024 (0.46)	996 (0.43)	9 (0.16)
Circulatory system									
Essential hypertension	1.28 (1.18, 1.38)***	70 (0.22)	1001 (0.22)	343 (0.14)	4 (0.06)	56 (0.19)	731 (0.17)	256 (0.11)	3 (0.05)
Heart valve disorders	1.18 (1.16, 1.20)***	1054 (3.33)	15,078 (3.24)	7584 (3.12)	97 (1.61)	899 (3.06)	11,970 (2.73)	6075 (2.64)	83 (1.43)
Acute cerebrovascular disease	1.39 (1.23, 1.57)***	32 (0.10)	413 (0.09)	173 (0.07)	3 (0.06)	22 (0.07)	269 (0.06)	130 (0.06)	3 (0.06)
Others									
Injuries^a^	1.18 (1.17, 1.18)***	13,453 (42.52)	16,4317 (35.28)	78,687 (32.37)	1292 (21.57)	10,493 (35.75)	130,995 (29.89)	63,419 (27.57)	990 (17.04)
Poisoning^a^	0.99 (0.92, 1.06)	123 (0.39)	1094 (0.23)	438 (0.18)	10 (0.17)	119 (0.41)	1089 (0.25)	372 (0.16)	15 (0.27)

Note: Age-adjusted frequency measures by SEP were calculated according the chronic or acute profile of each disease: ^a^Incidence proportion only for acute bronchitis, influenza, injuries, meningitis, poisoning and tuberculosis, or Period prevalence for the rest of diseases. (Incidence proportion accounts for the number of new cases of the disease and Period prevalence includes all cases of the disease (new and preexisting) during the period)

*ADHD* Attention deficit hyperactivity disorder, *ASD* Autism spectrum disorder

*p*-value: *(≤0.05), **(≤0.01), ***(≤0.001)

Of all the 29 pathologies studied, 25 were more frequent in boys. Autism spectrum disorder (ASD) (PR = 4.66, 95% CI: 4.39, 4.94), attention deficit hyperactivity disorder (ADHD) (PR = 2.70, 95% CI: 2.63, 2.76), meningitis (IR = 1.57, 95% CI:1.36, 1.80), and asthma (PR = 1.47, 95% CI:1.45, 1.49) were significantly more frequent in boys than in girls. By contrast, mood disorders (PR = 0.87, 95% CI: 0.83, 0.91) and dermatitis (PR = 0.94, 95% CI: 0.93, 0.94) were significantly more frequent in girls than in boys.

### Inequality measures

Measures of SEP inequality are presented in Table 3. Overall, 25 of the 29 diseases analysed showed a significant inverse SEP gradient: the lower the SEP, the higher the frequency. Only food allergy showed a significant direct SEP gradient (RII = 0.86, 95% CI: 0.82, 0.90 in boys and RII = 0.87, 95% CI: 0.83, 0.92). The other diseases studied (cancer of the brain and nervous system, leukaemia, cystic fibrosis for both sexes, and meningitis and acute cerebrovascular disease for girls) did not show statistical significance.

**Table 3:** Measures of SEP inequality SII and RII and sex differences of each disease in children in Catalonia

Disease	SII (95% CI)			RII (95% CI)		
	Boys	Girls	***p***-value*	Boys	Girls	***p***-value*
Infectious diseases						
Tuberculosis	0.08 (−0.04, 0.21)	0.11 (−0.13, 0.35)	0.675	8.13 (4.83, 13.71)	9.62 (5.70, 16.26)	0.656
Meningitis (bacterial or viral)	0.06 (−0.03, 0.15)	0.02 (− 0.03, 0.08)	0.144	1.89 (1.31, 2.72)	1.23 (0.77, 1.95)	0.153
Influenza	5.00 (−2.68, 12.67)	4.90 (− 2.47, 12.26)	0.969	1.29 (1.25, 1.33)	1.33 (1.28, 1.37)	0.286
Neoplasms						
Cancer of the brain and nervous system	0.04 (−0.04, 0.12)	− 0.03 (− 0.11, 0.05)	0.019	1.28 (0.75, 2.19)	1.13 (0.64, 2.02)	0.765
Leukaemia	−0.01 (− 0.07, 0.04)	0.01 (− 0.03, 0.05)	0.103	1.01 (0.65, 1.58)	1.16 (0.69, 1.94)	0.695
Malignant neoplasms	0.20 (−0.11, 0.50)	0.17 (− 0.06, 0.41)	0.793	1.37 (1.15, 1.62)	1.44 (1.19, 1.73)	0.696
Anthropometrics						
Overweight	11.36 (−0.81, 23.53)	10.69 (1.30, 20.08)	0.858	1.63 (1.58, 1.67)	1.78 (1.73, 1.84)	< 0.001
Obesity	7.98 (2.52, 13.43)	6.09 (2.35, 9.82)	0.261	2.37 (2.27, 2.48)	2.68 (2.54, 2.82)	< 0.001
Mental health						
Mood disorders	0.31 (0.14, 0.48)	0.44 (0.08, 0.81)	0.205	1.92 (1.65, 2.23)	2.09 (1.81, 2.41)	0.424
Adjustment and anxiety disorders	4.03 (0.66, 7.41)	4.03 (0.47, 7.60)	0.998	2.17 (2.07, 2.27)	2.24 (2.13, 2.36)	0.346
ADHD	1.48 (−0.39, 3.34)	0.60 (0.07, 1.13)	0.090	1.21 (1.15, 1.27)	1.31 (1.20, 1.43)	0.112
ASD	0.53 (−0.31, 1.37)	0.15 (−0.09, 0.40)	0.107	1.29 (1.16, 1.42)	1.36 (1.09, 1.71)	0.636
Autoimmune and respiratory diseases						
Acute bronchitis	8.62 (−3.28, 20.52)	8.19 (−3.40, 19.77)	0.915	1.35 (1.33, 1.38)	1.39 (1.36, 1.42)	0.090
Chronic bronchitis	4.46 (−1.75, 10.67)	4.13 (−1.62, 9.89)	0.874	1.37 (1.33, 1.41)	1.43 (1.38, 1.49)	0.064
Asthma	3.00 (−2.63, 8.64)	2.68 (− 1.42, 6.78)	0.848	1.08 (1.05, 1.12)	1.23 (1.18, 1.29)	< 0.001
Respiratory allergy	2.71 (−0.33, 5.75)	2.58 (−0.03, 5.18)	0.889	1.26 (1.22, 1.29)	1.37 (1.33, 1.41)	< 0.001
Dermatitis	10.68 (−3.47, 24.84)	12.55 (−2.75, 27.86)	0.713	1.32 (1.30, 1.35)	1.41 (1.38, 1.43)	< 0.001
Food allergy	−0.06 (−1.80, 1.67)	−0.23 (−2.15, 1.68)	0.791	0.86 (0.82, 0.90)	0.87 (0.83, 0.92)	0.655
Cystic fibrosis	0.00 (−0.03, 0.02)	−0.02 (− 0.05, 0.02)	0.113	0.79 (0.40, 1.55)	0.77 (0.38, 1.55)	0.963
Adverse birth outcomes						
Short gestation, low birth weight, foetal growth retardation.	0.53 (0.42, 0.65)	0.54 (0.39, 0.70)	0.788	2.20 (1.99, 2.43)	2.14 (1.92, 2.38)	0.704
Intrauterine hypoxia and birth asphyxia and trauma	0.36 (0.11, 0.61)	0.28 (−0.09, 0.65)	0.453	2.31 (1.98, 2.69)	2.31 (1.93, 2.78)	0.978
Congenital anomalies	5.21 (−2.26, 12.69)	3.59 (−3.77, 10.96)	0.530	1.38 (1.34, 1.42)	1.23 (1.19, 1.27)	< 0.001
Nervous system						
Paralysis	0.14 (−0.02, 0.30)	0.11 (− 0.12, 0.33)	0.654	1.89 (1.48, 2.41)	1.66 (1.26, 2.2)	0.501
Epilepsy	0.18 (−0.17, 0.53)	0.34 (−0.21, 0.88)	0.312	1.47 (1.29, 1.68)	1.30 (1.13, 1.50)	0.203
Circulatory system						
Essential hypertension	0.15 (0.07, 0.24)	0.13 (0.06, 0.19)	0.284	2.14 (1.72, 2.68)	2.16 (1.66, 2.79)	0.978
Heart valve disorders	1.40 (−1.40, 4.20)	1.27 (−0.96, 3.50)	0.885	1.17 (1.11, 1.23)	1.21 (1.15, 1.29)	0.318
Acute cerebrovascular disease	0.04 (0.01, 0.06)	0.01 (−0.02, 0.05)	0.039	1.72 (1.23, 2.38)	1.26 (0.85, 1.86)	0.232
Others						
Injuries	16.35 (−5.98, 38.67)	14.63 (− 5.84, 35.10)	0.815	1.31 (1.29, 1.33)	1.29 (1.28, 1.31)	0.242
Poisoning	0.16 (−0.23, 0.56)	0.11 (− 0.43, 0.65)	0.741	2.02 (1.65, 2.48)	2.51 (2.03, 3.10)	0.150

*ADHD* attention deficit hyperactivity disorder, *ASD* autism spectrum disorder

*p-value to test sex differences for SII and RII (sex differences were assessed by inserting a two-way interaction term between SEP and sex)

**Fig. 1:**
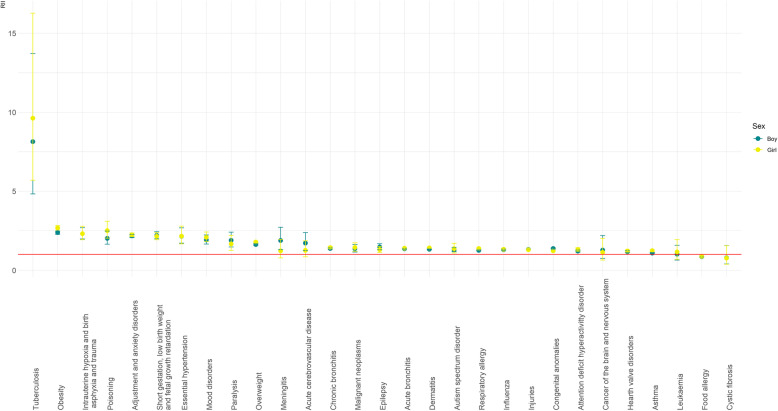
RII for each disease by sex in the population under 15 in Catalonia

The diseases with a more pronounced SEP gradient (RII ≥ 2.0) in both sexes were TB, obesity, adjustment and anxiety disorders, essential hypertension, poisoning, short gestation, low birth weight and foetal growth retardation, and intrauterine hypoxia and birth asphyxia and trauma. Following these closely, but with slight differences depending on sex, were mood disorders, meningitis and paralysis in boys, and mood disorders and overweight in girls (see Fig. 1).

Regarding the differences between sex in inequalities, relative inequalities were significantly higher in girls than in boys for respiratory allergy, asthma, dermatitis, being overweight, and obesity (*p* < 0.05). In contrast, boys showed significantly higher relative inequalities compared to girls only in congenital anomalies (*p* < 0.05). Absolute inequalities were significantly greater in boys compared to girls for acute cerebrovascular disease and cancer of the brain and nervous system (*p* < 0.05).

### Population SEP impact measures

The population SEP impact measures are presented in Table 4. The diseases that could potentially be prevented more from equating the health status of the childhood population to the medium-high SEP were, in relative terms, TB (PAF% = 56.26, 95% CI: 43.03, 66.41 in boys and PAF% = 51.95, 95% CI: 38.51, 62.45 in girls), intrauterine hypoxia and birth asphyxia and trauma (PAF% = 30.04, 95% CI: 25.40, 34.39 in boys and PAF% = 32.10, 95% CI: 26.54, 37.24 in girls), obesity (PAF% = 27.14, 95% CI: 25.86, 28.39 in boys and PAF% = 30.31, 95% CI: 28.77, 31.80 in girls), and short gestation low birth weight and foetal growth retardation (PAF% = 29.83, 95% CI: 26.78, 32.75 in boys and PAF% = 28.21, 95% CI: 24.98, 31.29 in girls). In absolute terms, the diseases or risk factors with the highest potential of preventable individuals affected due to the impact of SEP were dermatitis (PAFa = 15,345, 95% CI: 14457, 16,227 in boys and PAFa = 19,027, 95% CI: 18128, 19,921 in girls), injuries (PAFa = 19,432, 95% CI: 18303, 20,555 in boys and PAFa = 14,758, 95% CI: 13714, 15,796 in girls), acute bronquitis (PAFa = 14,082, 95% CI: 13174, 14,938 in boys and PAFa = 11,905, 95% CI: 11091, 12,713 in girls), and being overweight (PAFa = 10,905, 95% CI: 10280, 11,524 in boys and PAFa = 10,872, 95% CI: 10299, 11,438 in girls).

**Table 4:** Measures of SEP impact PAF% and PAFa of each disease by sex in children in Catalonia

Disease	PAF% (95% CI)		PAFa (95% CI)	
	Boys	Girls	Boys	Girls
Infectious diseases				
Tuberculosis	56.26 (43.03, 66.41)	51.95 (38.51, 62.45)	181 (139, 214)	171 (127, 205)
Meningitis (bacterial or viral)	18.32 (5.88, 29.11)	7.69 (−9.09, 21.90)	93 (30, 148)	24 (−28, 67)
Influenza	7.17 (6.13, 8.20)	7.83 (6.73, 8.91)	4540 (3882, 5191)	4450 (3827, 5065)
Neoplasms				
Cancer of the brain and nervous system	0.54 (−19.66, 17.34)	6.69 (−14.89, 24.21)	1 (−44, 39)	13 (−29, 47)
Leukaemia	−1.77 (− 18.54, 12.63)	5.93 (− 13.30, 21.90)	− 6 (− 60, 41)	14 (− 32, 53)
Malignant neoplasms	7.51 (1.57, 13.10)	9.18 (2.85, 15.10)	166 (35, 290)	177 (55, 291)
Anthropometrics				
Overweight	15.63 (14.74, 16.52)	18.44 (17.46, 19.40)	10,905 (10,280, 11,524)	10,872 (10,299, 1438)
Obesity	27.14 (25.86, 28.39)	30.31 (28.77, 31.80)	9407 (8694, 9841)	7204 (6840, 7560)
Mental health				
Mood disorders	20.11 (15.29, 24.65)	21.54 (16.96, 25.86)	619 (471, 759)	718 (565, 862)
Adjustment and anxiety disorders	22.88 (21.42, 24.31)	23.30 (21.76, 24.81)	6964 (6520, 7400)	6290 (5873, 6699)
ADHD	4.01 (2.31, 5.68)	7.34 (4.46, 10.13)	1031 (595, 1459)	658 (399, 908)
ASD	7.04 (3.63, 10.33)	8.57 (0.84, 15.69)	459 (237, 673)	113 (11, 207)
Autoimmune and respiratory diseases				
Acute bronchitis	10.30 (9.63, 10.96)	11.11 (10.35, 11.87)	14,082 (13,174, 14,983)	11,905 (11,091, 12,713)
Chronic bronchitis	10.30 (9.27, 11.32)	11.52 (10.35, 12.68)	6540 (5887, 7187)	5665 (5089, 6234)
Asthma	1.15 (0.03, 2.31)	5.16 (3.69, 6.61)	594 (16, 1196)	1714 (1225, 2194)
Respiratory allergy	6.93 (6.01, 7.83)	9.59 (8.59, 10.58)	2335 (2026, 2641)	2295 (2056, 2532)
Dermatitis	9.69 (9.13, 10.25)	11.61 (11.06, 12.15)	15,345 (14,457, 16,227)	19,027 (18,128, 19,921)
Food allergy	−5.42 (−7.07, −3.80)	−4.46 (−6.22, −2.73)	− 524 (− 683, − 367)	− 401 (− 559, − 246)
Cystic fibrosis	−5.12 (− 32.35, 16.51)	−8.90 (− 37.10, 13.50)	−7 (− 43, 22)	−11 (− 47, 17)
Adverse birth outcomes				
Short gestation, low birth weight,foetal growth retardation	29.83 (26.78, 32.75)	28.21 (24.98, 31.29)	2053 (1843, 2254)	1757 (1556, 1949)
Intrauterine hypoxia and birth asphyxia and trauma	30.04 (25.40, 34.39)	32.10 (26.54, 37.24)	915 (774, 1048)	675 (558, 783)
Congenital anomalies	11.51 (10.55, 12.45)	8.19 (7.10, 9.26)	8305 (7615, 8988)	4742 (4115, 5362)
Nervous system				
Paralysis	18.50 (10.39, 25.88)	11.37 (1.81, 20.00)	211 (118, 295)	98 (16, 173)
Epilepsy	11.22 (6.86, 15.38)	6.11 (1.21, 10.78)	443 (270, 607)	196 (39, 346)
Circulatory system				
Essential hypertension	24.81 (17.73, 31.28)	24.64 (16.36, 32.10)	351 (251, 443)	257 (171, 335)
Heart valve disorders	5.62 (3.87, 7.34)	6.39 (4.43, 8.31)	1340 (921, 1750)	1218 (844, 1584)
Acute cerebrovascular disease	17.09 (5.93, 26.93)	7.09 (−6.98, 19.31)	107 (37, 169)	30 (−30, 82)
Others				
Injuries	7.54 (7.10, 7.98)	7.17 (6.67, 7.68)	19,432 (18,303, 20,555)	14,758 (13,714, 15,796)
Poisoning	20.81 (14.22, 26.90)	27.82 (21.26, 33.84)	347 (237, 449)	442 (337, 537)

Note: PAF% and PAFa were calculated using medium-high SEP category as a reference group

*ADHD* attention deficit hyperactivity disorder, *ASD* autism spectrum disorder

In terms of population impact, 7.80% (95% CI: 7.53, 8.06) of the overall disease events analysed in boys and 8.88% (95% CI: 8.58, 9.17) in girls would be avoided if all children had the same disease rates as the medium-high SEP group. That is translated into 80,188 disease events (95% CI: 77456, 82,912) in boys and 74,921 disease events (95% CI: 72429, 77,405) in girls that would be potentially preventable (data for the total burden of diseases studied; for the PAF results of each disease, see Table 4). By contrast, food allergy showed a significant negative attributable population impact (PAF% = − 5.42, 95% CI: − 7.07, − 3.8 in boys and PAF% = − 4.46, 95% CI: − 6.22, − 2.73 in girls).

**Fig. 2:**
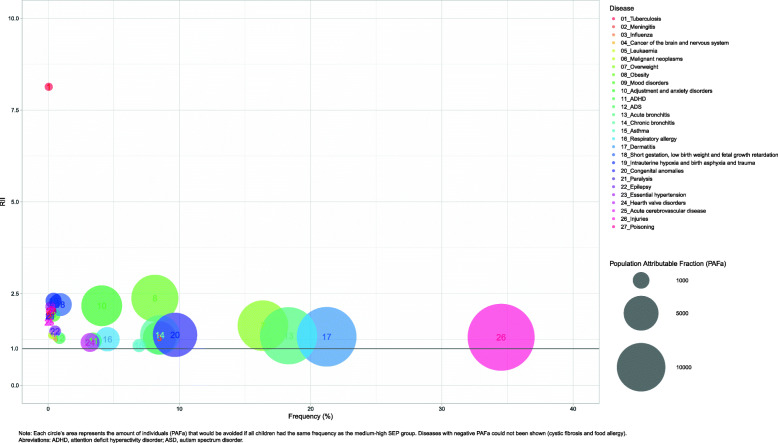
RII (y axis), frequency (x axis) PAFa (bubble) for each disease in boys population under 15 years old in Catalonia

**Fig. 3:**
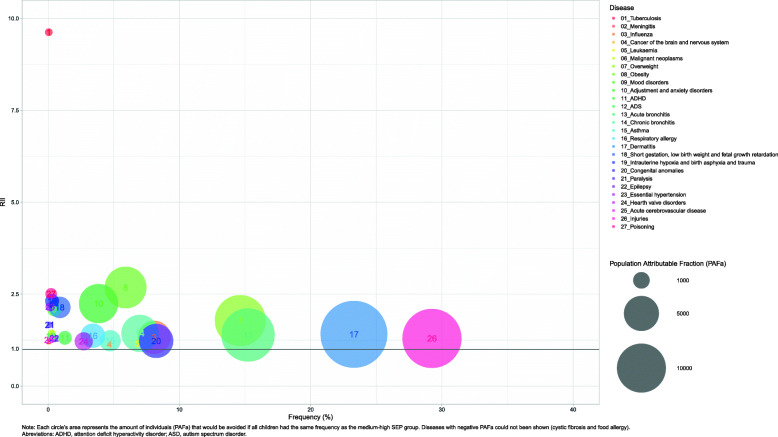
RII (y axis), frequency (x axis) PAFa (bubble) for each disease in girls population under 15 years old in Catalonia

Figure 2 for boys and Fig. 3 for girls show three different inequality measures for each disease and sex.

## Discussion

### Main findings

This study confirms that socioeconomic inequalities are present in a large number of diseases of different etiologies and levels of seriousness (from malignant neoplasms to dermatitis), in the entire population of children in Catalonia. The systematic pattern of socioeconomic inequality observed highlights that, beyond the nature and idiosyncrasy of each disease, 1) the entire child population is affected by this social phenomenon, 2) SEP plays a common role in their development, and 3) gender differences in inequalities exist in different diseases and risk factors. This is consistent with the findings of different studies where social class and child health status were assessed [6, 7, 9, 27, 35].

To calculate health inequalities and to enrich later interpretations and findings, we calculated four summary measures of health inequalities between SEP levels in each disease and the burden of the diseases attributable to SEP. The purpose was to capture both absolute and relative inequality measures accounting for all SEP groups and their size in the studied population. On the other hand, absolute measures provided the total number of disease events affected by SEP and relative measures tend to quantify the association of SEP and the outcome. Summary measures, based on regression, take into account all SEP groups weighted according to their real size rather than an equal distribution. Therefore, they provided a gradient that includes the entire population, being less biased than using pairwise measures in which only very low and high SEP would be taken into account. This last approach could be more affected by undereported health outcomes, mainly in the high SEP group, due to their use of private healthcare services [26, 29].

### Comparison with other studies and interpretation of the results

SEP, as a social determinant of health, influences the distribution of diseases among the population, as shown in this and previous studies [6, 27, 36]. Although the pathways by which SEP impacts on health are mostly indirect, SEP influences factors such as nutrition, housing conditions, and education, hence the degree of vulnerability and social exclusion of children [1].

Social vulnerabilities in childhood is a broad concept that includes all the negative experiences that could affect children’s development and, therefore, their health. However, income level could exacerbates or buffer the effect of the social vulnerabilities in lifestyle and health outcomes [1, 7]. All these social experiences in childhood impact on a crucial period of development through different complex and inter-related pathways such as biological embedding, material deprivation, psychosocial or structural mechanisms. All these pathways, highly dependent on the family’s relative SEP, act simultaneously creating social gradients in present and future health outcomes [1, 37].

The results observed in this study indicate that the gradients between SEP and health present different intensities depending on the disease. This sheds more light on the identification of those diseases with a high social factor in their construction and distribution. In concordance with other studies, the diseases that show the greatest inequalities are TB, obesity, hypertension, adverse birth outcomes, and mental health disorders [27, 38–41]. TB is by far the disease with most inequalities in Catalonia and elsewhere [38]. TB is related to poverty (children from very low SEP levels are at least up to 8 times more likely to have it than their most advantaged peers) and it is particularly exacerbated in immigrant populations [42, 43]. Screening programs and an improvement in working and housing conditions stand out as the greatest contributions to eradicating TB [38]. As in other studies which test inequalities in different health outcomes, obesity (the most acute form of overweight) and hypertension, both related to lifestyles, show a high association with SEP [27]. There is a lot of evidence which shows that diseases related to lifestyles are greatly impacted by social determinants such as SEP or a parent’s educational level, or nutrition and physical activity, among others [15, 16, 27, 39]. Therefore, within a global upward trend, the number of children who suffer from this inequality is also increasing [17].

Special attention should be paid to inequalities in adverse birth outcomes. As our results reveal, they are broadly present [40] or even increase in countries similar to Catalonia [44]. These outcomes are related to pregnancy and delivery, which are periods of intense surveillance by the healthcare system. This situation emphasizes that other factors such as employment status or SEP level (both individual and neighbourhood) are key in explaining these inequities beyond the responsability of the health system [40, 44]. Regarding mental health disorders, our findings show that SEP inequalities stand out in concordance with other studies [6, 27, 41]. Other factors as mental health or the employment status of parents (mainly of mothers) also play an important role [28] being particularly incisive in contexts of economic crisis [18]. Significant attention must be given to those diseases that would potentially be most reduced by improving children’s SEP. These diseases are not those showing the highest inequalities but those that account for the largest number of preventable disease events (mainly due to their high prevalence in childhood population), namely: dermatitis, injuries, acute bronchitis, obesity, and being overweight.

Previous studies on children have not taken into account gender as an axis of inequality, assuming that the social role of gender does not influence health inequalities in childhood. By contrast, our study has shown differences in many of the measures calculated. In general terms, the PR or IR of boys compared to girls is higher, to the boys’ detriment. This finding is consistent with the slightly increased vulnerability in boys (focused in the early ages) and the physiological differences between the sexes [21, 45]. However, in terms of inequalities, this study has found that girls suffer from significant differences in inequalities in more pathologies in comparison to boys. This finding points to an intersectionality between sex and SEP where the two axes of inequality jointly create roles of multiplicative discrimination and subordination [46].

This study brings attention to a possible gender bias in paediatrics healthcare. Gender bias in paediatrics involves a number of different actors: the infant as a patient, the parents who present the child’s symptoms, and the medical professional who interprets the patient’s narratives and behaviours. Several studies have revealed that girls versus boys receive less effort to obtain an advanced diagnostic, a therapeutic intervention, or an adequate pain assessment, have different hospitalisation rates for the same conditions and experience more preconceptions about pathology patterns [22, 23, 47, 48]. SEP, sex, gender bias, and their intersections simultaneously influence the process of disease construction via diverse pathways and in differing intensities [21, 24]. Gender bias is well known in adulthood but little research has been done on the phenomenon in childhood, as if it did not exist [31, 49]. Future research should explore further on gender-related health inequalities in childhood.

With regard to food allergy, other studies have also highlighted its direct SEP gradient [27]. An increasing global trend awareness and the widespread screening of food allergy supports our finding of a direct SEP gradient [50]. High-SEP parents tend to be more concerned with identifying food allergies in their children and seek out more tests than the lowest-SEP parents, contributing to overdiagnosis in socioeconomically advantaged children [51]. Other studies also add that part of these socioeconomic differences may be caused by underdiagnosis in disadvantaged groups [27], eventhough in our study population low SEP children do a higher number of visits to the healthcare system than their advantatged peers [20].

Our results confirm that, despite the important role of universal healthcare coverage in guaranteeing free and equal access to the health system, it does not assure achieving equity in health status. Thus, similar findings have encouraged governments to include social determinants as a main cause of health inequalities [9].

### Strengths and weaknesses of the study

This study included a broad representation of the childhood pathology; 29 diseases or risk factors of different etiologies, magnitudes, seriousness, and affecting diverse systems of the human body. It provides high-value evidence of the broad impact of social determinants on health and a useful variety of inequality measures, absolute and relative, with the intention of influencing population and political decisions.

Calculating the same inequality measures in a broad set of diseases on the same population increases the robustness of comparisons between diseases as an homogeneous methodology has been used, and as all the population is affected by the same common external factors.

This population-based study included the total population under 15 years old in Catalonia, nearly 1,500,000 individuals over a 4-year period providing a large capacity to detect more individuals with low-prevalence diseases.

The study used individual-level data, which allowed economic and employment information for one of a child’s guardians to be linked with the child’s health status. However, this data does not provide complete information about household economic status or a more detailed segmentation of the SEP variable but it has been used successfully in different studies [20, 28].

The SEP indicator used is based on the parent’s economic and employment situation, which is available from the Catalan Department of Health databases. Other commonly used indicators such as parental education or occupation were not available. Besides, income has proven to have a strong association with health and to influence a wide range of material circumstances with direct implications for health [52].

Medical diagnoses are based on contacts with the public healthcare system. Even where universal healthcare access is granted, private and public health services co-exist, especially for high SEP families. It is estimated that 30% of all children under the age of 15 use both private and public healthcare (88% from medium and high SEP) [53]. This can lead to an underreporting of some diseases, especially those among the most frequent, less severe and acute profile, such as respiratory diseases or dermatitis.

## Conclusion

Although health inequalities will be difficult to eliminate entirely, efforts must be made in all institutions to work actively toward eradicating such social injustice in order to accomplish one of the SDGs, namely “Reduce inequality within and among countries”, by 2030 [10]. In the current time, when we are facing a new economic crisis resulting from the COVID-19 pandemic, which will likely increase poverty and socioeconomic inequalities, this goal has only increased in importance.

Socioeconomic inequalities in childhood not only imply unequal health outcomes but also inequalities in other indicators of wellbeing, educational attainment, and future career opportunities. It is clear that more attention is required in childhood to tackle the vicious intergenerational circle of poverty and social exclusion that begins in childhood [2].

Policies and programs focused on improving family’s childhood context (i.e housing, nurturing, parent’s behaviours), specially for the most vulnerable, have been identified as the most efficient ways to reduce health inequalities in childhood and achieve a fairer society [3, 4].

## Supplementary Information

**Additional file 1.**

## Data Availability

CMBD is an administrative registry constructed and fed with the information that all centres send in to the Administration. The Administration automatically dissociates any personal information from the rest, and assigns a randomly generated number to each case. This register is used by the Administration to plan healthcare services, and to assess the quality of the care provided. Using this register, AQuAS each year publishes a set of 100 indicators (at aggregated level) to evaluate the quality of the care provided by Catalan healthcare centres. This information is publicly available for citizens, professionals and managers at http://observatorisalut.gencat.cat/en/index.html. The anonymised and unidentified data at individual level will be accessible to the research staff of the research centres accredited by the Research Centres of Catalonia (CERCA) institution, SISCAT agents, and public university research centres, as well as the same health administration.

## Abbreviations

SEP: Socioeconomic position
SDG: Sustainable Development Goal
RII: Relative index of inequality
Catalan acronym RCA: Central Registry of Insured Persons
Catalan acronym CMBD: Registry of the Minimum Basic Dataset
TB: Tuberculosis
PR: Prevalence ratio
IR: Incidence proportion ratio
PAF%: Population attributable fraction 21
SII: Slope index of inequality
PAFa: Attributable fraction absolute
ASD: Autism spectrum disorder
ADHD: Attention deficit hyperactivity disorder
