# Supplementary material for: Socioeconomic inequalities in 29 childhood diseases: evidence from a 1,500,000 children population retrospective study
Source: BMC Public Health. 2021 Jun 16;21:1150. doi: 10.1186/s12889-021-11230-9 (PMC8205646; doi:10.1186/s12889-021-11230-9)
Supplement: Supplementary file 1 — Additional file 1. [file 12889_2021_11230_MOESM1_ESM.docx]

**Additional file 1. Clinical pathology codes included in each disease.**

| **Disease variable** | **CCS** | **ICD-9-MC** |
| --- | --- | --- |
| Acute bronchitis |  | 466, 466.0, 466.1, 466.11, 466.19, 519.11 |
| Acute cerebrovascular disease | 109 |  |
| Attention deficit hyperactivity disorder |  | 314.00, 314.01 |
| Adjustment and anxiety disorders | 650, 651 |  |
| Asthma | 128 |  |
| Autism spectrum disorder |  | 299.0, 299.00, 299.01 |
| Cancer of brain and nervous system | 35 |  |
| Chronic bronchitis | 127 |  |
| Congenital anomalies | 213-217 |  |
| Cystic fibrosis | 56 |  |
| Dermatitis |  | 691, 692, 708, 691.0, 691.8, 692.0, 692.1, 692.2, 692.3, 692.4, 692.5, 692.6, 692.7, 692.8, 692.9, 708.0, 708.1, 708.2, 708.3, 708.4, 708.5, 708.8, 708.9, 692.70, 692.71, 692.72, 692.73, 692.74, 692.79, 692.81, 692.82, 692.83, 692.84, 692.89 |
|  |  | Children with food or respiratory allergy not included. |
| Epilepsy | 83 | Not included 780.3 |
| Essential hypertension | 98 |  |
| Food allergy |  | 693, 477.1, 558.3, 693.0, 693.1, 693.8, 693.9, 995.6, 995.7, 995.60, 995.61, 995.62, 995.63, 995.64, 995.65, 995.66, 995.67, 995.68, 995.69, 579.0 |
| Heart valve disorders | 96 |  |
| Influenza | 123 |  |
| Injuries | 225-236, 239-240, 244 |  |
| Intrauterine hypoxia and birth asphyxia and trauma | 220, 223 |  |
| Leukemia | 39 |  |
| Malignant neoplasms | 11-45 |  |
| Meningitis | 76 |  |
| Mood disorders | 657 |  |
| Paralysis | 82 |  |
| Poisoning | 241-243 |  |
| Respiratory allergy |  | 518.6, 477, 477.0, 477.2, 477.8, 477.9 |
|  |  | Children with food allergy not included. |
| Short gestation, low birth weight and fetal growth retardation | 219 |  |
| Tuberculosis | 1 |  |
| Obesity | >+2SD (more than two standard deviation above the median in WHO growth reference for children and adolescents)^α^ | |
| Overweight | >+1SD (more than one standard deviation above the median in WHO growth reference for children and adolescents)^α^ | |

^α^WHO. Growth reference data for 5-19 years old. Available: <https://www.who.int/tools/growth-reference-data-for-5to19-years>

Abreviations: CCS Clinical Classifications Software, ICD-9-MC International Classification of Diseases, 9th revision, Clinical modification, SD Standard deviation
